# Sensitive Aflatoxin B1 Determination Using a Magnetic Particles-Based Enzyme-Linked Immunosorbent Assay

**DOI:** 10.3390/s8127571

**Published:** 2008-11-26

**Authors:** Madalina Tudorache, Camelia Bala

**Affiliations:** 1 Laboratory of Quality Control and Process Monitoring and University of Bucharest, 4-12 Regina Elisabeta, 030018 Bucharest, Romania; E-mail: madalina.sandulescu@g.unibuc.ro; 2 Department of Analytical Chemistry, University of Bucharest, 4-12 Regina Elisabeta, 030018 Bucharest, Romania; E-mail: madalina.sandulescu@g.unibuc.ro

**Keywords:** Magnetic particle-based enzyme-liked immunosorbent assay (*mp*-ELISA), Aflatoxin B1, immobilization, antibody, sensitivity

## Abstract

A magnetic particle-based enzyme-liked immunosorbent assay (*mp*-ELISA) has been developed as new an alternative immunoassay for Aflatoxin B1 determination. The method is based on conventional competitive ELISA whereby the anti-Aflatoxin B1 antibody is immobilized on the magnetic particles' surface. The influence of the antibody type as well as antibody immobilization on the magnetic beads surface was investigated in detail. Also, optimum values for the general parameters of the method (e.g. tracer concentration, type of antibody, and incubation time) were established. Finally, a sensitive immunoassay method (*mp*-ELISA) was performed for Aflatoxin B1 determination at ppt level (LOD = 1 ppt Aflatoxin B1).

## 1. Introduction

Aflatoxin B1, like the other aflatoxins (e.g. aflatoxin M1, G1, B2 and G2) is one of the most frequent mycotoxins occurring on agricultural commodities in the field and during storage under a wide range of climatic conditions and/or during technical transformation and preparation of food [1]. Aflatoxin B1 could also very easily contaminate food and feed products (e.g. milk, animal tissue, eggs, *etc.*) by “carry over”. It is potent toxin with severe effects on animal and human health, e.g. cyto-, nephro- or neurotoxic, carcinogenic, mutagenic, immunosuppressive and estrogenic effects [2, 3]. Therefore, special attention has been accorded to Aflatoxin B1, especially in feed. Directive 2003/100/EC of 31 October 2003 on Undesirable Substances in Animal Feed set limits for Aflatoxin B1 in terms of mg/kg of feed with regard both to raw materials and feed of different composition (compound, complete and complementary feed) [4]. The maximum level of Aflatoxin B1 was set at 2 μg/kg (max.) of Aflatoxin B1 especially in groundnuts, nuts, dried fruits and cereals [5].

The requirement to apply this regulatory limit has prompted the development of a vast number of analytical methods for the qualitative and quantitative determination of Aflatoxin B1 in different samples, such as food, feed, and other biological materials. Thus, current analysis methods are thin-layer chromatography (TLC) [6, 7], liquid chromatography and high-performance liquid chromatography (HPLC) [8, 9], immunochromatography [10], electrochemical immunoanalysis [11, 12] and microtitre plate enzyme-linked immunosorbent assay (ELISA) [9, 13, 14]. All of those methods allow detection of Aflatoxin B1 over a large concentration range (i.e. from ppt to ppm level). Unfortunately, they require long analysis time, high performance of the equipment and operating personnel and are very costly.

In order to eliminate such inconveniences, we propose a new immunoassay for Aflatoxin B1 determination called magnetic particle-based enzyme-liked immunosorbent assay (*mp*-ELISA). The method principle is based on conventional competitive ELISA whereby the anti-Aflatoxin B1 antibody is immobilized on the magnetic particles' surface. A permanent magnet placed close to the microplate walls allows easy manipulation of the magnetic particles together with the immobilized reagent (e.g. anti-Aflatoxin B1 antibody). Therefore, the antibody-particles are easily dispensed in the reaction phase and also precipitated on the well bottom. We tested the influence of the antibody immobilization procedure as well as the antibody type on the *mp*-ELISA sensitivity. The method was applied for Aflatoxin B1 determination using optimized parameters.

## 2. Results and Discussion

The principal feature of *mp*-ELISA is the use of magnetic particles as solid support for immunoreagent immobilization (anti-Aflatoxin b1 antibody). Magnetic particles are micro/nano size spheres of iron oxide (Fe_3_O_4_ or y-Fe_2_O_3_) covered with a polymeric material, which allows physical/ covalent/affinity attachment, usually of the antibody (Ab), onto the particle surface (Ab-bead) [18]. Despite their name *magnetic particles*, many of the commercial beads are superparamagnetic, meaning that these can immediately be magnetised with an external magnetic field and redispensed immediately once the magnet is removed [19]. The particles' size (micro/nanometer in diameter) allows the possibility of handling the particles in suspension (avoiding the self-aggregation of magnetic particles!).

Therefore, by using a magnetic field the Ab-bead can be easily located in the expected/needed place of the reaction vial avoiding the kinetic limitations of the process due to reagent diffusion, a frequent problem of the ELISA (enzyme-linked immunosorbent assay) approach. Also, a *comfortable* separation of bound and free fraction of the tracer can be performed only through a simple collection of the magnetic particles by a permanent magnet [16, 17]. Other advantages of magnetic beads compared with the conventional solid supports used in immunoassays (e.g. ELISA microtitre plate, polymer particles, cellulose membrane, *etc.*) are easy manipulation, low pressure drop, high mass transfer rate, good fluid-solid contact, perspectives for system automation and miniaturization [19-21].

Thus, an improvement of the ELISA concept had been released using the magnetic particles as solid support of the antibody (*mp*-ELISA). The method was optimized by determining the best values for parameters such as type of antibody, immobilization procedure of antibody, tracer concentration and incubation time corresponding to immunoaffinity interaction.

### 2.1. Antibody immobilization on the magnetic particles

The antibody will be immobilised on the magnetic surface. Different immobilisation chemistries will be tested, such as covalent and affinity immobilization, in order to obtain an efficient coverage of the magnetic particle surface with the antibody, and also to avoid the blocking the antibody's active sites. In the first case, the antibody was attached covalently via –COOH groups grafted on the particle surface and the –NH_2_ groups of the antibody structure. In the second case the pre-coverage of the particles surface with protein A/G was assumed. Then, the antibody was easily recognized and captured on the particles surface since protein A/G has high affinity for Fc part of the antibody [22].

A briefly study of the specific literature demonstrated that there is still no consensus regarding the most efficient antibody immobilisation procedure. The explanation could be that each antibody is a unique entity and its behaviour after immobilisation cannot be rigorously predicted. Therefore, we decided to test different types of antibody for each immobilization case (Ab I – polyclonal Ig G anti-Aflatoxin B1 antibody, Ab II – affinity purified polyclonal Ig G anti-Aflatoxin B1 antibody, and Ab III – monoclonal Ig G anti-Aflatoxin B1 antibody). A study was also performed on the antibody-tracer immuno-interaction knowing that the antibody might recognize both tracer and analyte in a similar way.

**Figure 1.**
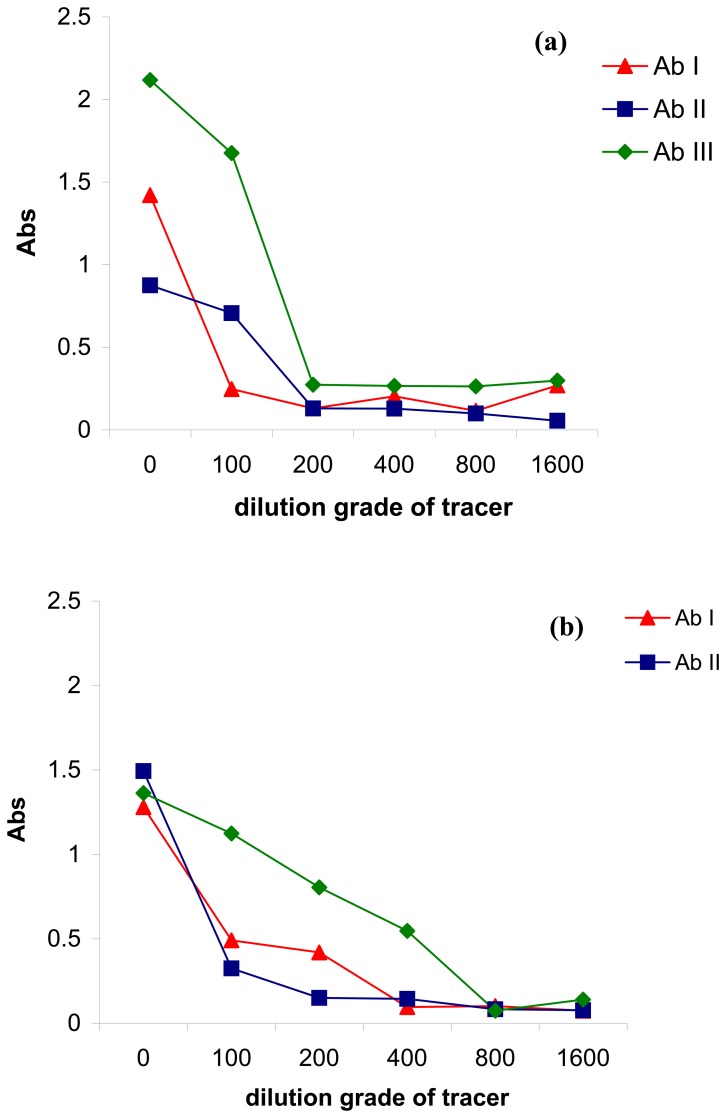

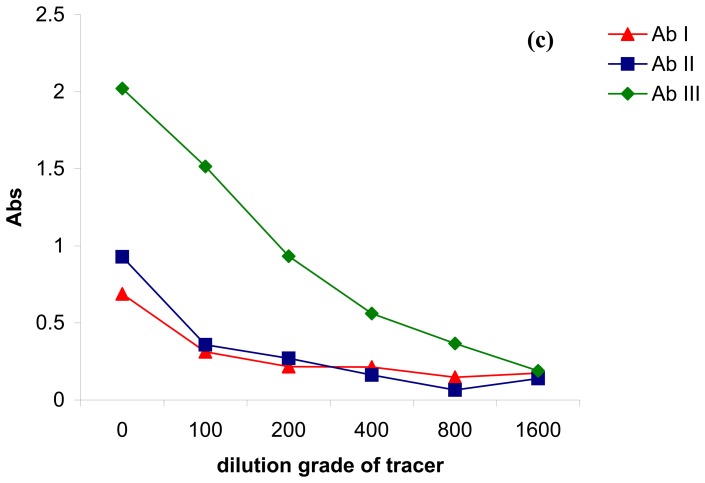
Dilution curve of tracer in the case of (a) covalent immobilization of antibody on the magnetic particles surface; (b) affinity immobilization of antibody on the magnetic particles surface *via* protein G; (c) affinity immobilization of antibody on the magnetic particles surface *via* protein A (1:1000 dilution of antibody-beads suspension).

Figure 1a shows the antibody behavior when the covalent immobilization procedure was applied. Ab III gave the highest signal when undiluted tracer solution was used. Therefore, a higher amount of Ab III was immobilized, compared with Ab I and Ab II. Also, if we take in consideration the slope of the curves (in the dynamic range), Ab III affinity for tracer seems to be higher than in the case of Ab I and Ab II (i.e. higher slope of Ab III curve than Ab II and Ab I slope).

When the magnetic particles were covered with protein G (Figure 1b), all of the antibodies (Ab I, II and III) gave similar signal corresponding to undiluted tracer solution. Therefore, protein G apparently recognized the antibodies in the same way, but the slope of the immuno-interaction tracer-antibody is lower for Ab III, which indicated that the antibody affinity was affected by the immobilization procedure (compared with the previous case). For affinity immobilization based on protein A (protein A) (Figure 1c), the antibodies behaved in a similar way with the case of covalent immobilization (Figure 1a).

As a general feature, Ab III was immobilized better, compared with Ab I and Ab II in all the cases, so Ab III was chosen as optimum immunoreagent for further experiments. Based on these experiments, the optimum tracer concentration was set up at 1:100 dilution grade of tracer for which the analytical signal was high enough to be used for further analysis. Also, 10 min incubation time of the immuno-affinity interaction antibody-tracer was used for all the experiments since that was enough for the antibody to recognize and bind the specific tracer (data not included in this paper).

### 2.2. Calibration of mp-ELISA method for Aflatoxin B1 determination

**Figure 2.**
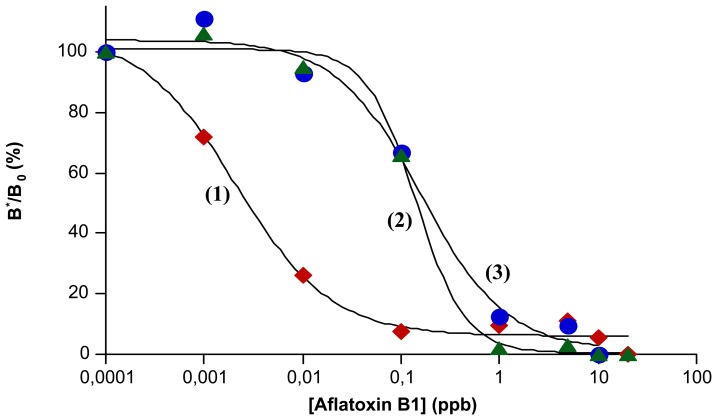
Dilution curves of Aflatoxin B1 for different immobilization procedures of the antibody on the magnetic particles surface: (1) covalent immobilization; (2) affinity immobilization via protein A; (3) affinity immobilization via protein G. Experimental conditions: 1:1000 dilution grade of Ab III-bead suspension and 1:100 dilution grade of tracer). (B*/B_0_ is the rate of the signal corresponding to bond fraction of the tracer and zero dose.)

A dilution curve of Aflatoxin B1 using *mp*-ELISA method was performed for any immobilization procedure (Figure 2). All configurations of the immobilized Ab III on the magnetic particles surface were tasted in terms of antibody-analyte interaction. There is an evident difference between *mp*-ELISA with antibodies immobilized by covalent and affinity interaction (Figure 2). On the other hand, protein A and protein G seems to afford similar performance of the method (Figure 2). Therefore, the best sensitivity of *mp*-ELISA method was obtained when Ab III was covalent attached on the beads (LOD = 1 ppt) (see Table 1). The affinity binding of Ab III gave also good sensitivity (LOD = 50 ppt and 100 ppt for the case of protein G and A, respectively), but lower that previous case (see Table 1). As we can see, *mp*-ELISA allows the determination of Aflatoxin B1 at the ppt level. Thus, *mp*-ELISA is one of the most sensitive methods for Aflatoxin B1 analysis, comparable with electrochemical biosensors (LOD = 30 ppt and 90 ppt) [23, 24] and classical direct competitive ELISA (LOD = 50 ppt and 4 ppt) [14, 25], which are currently the best.

## 3. Experimental Section

### 3.1. Chemicals and instruments

Aflatoxin B1 (Sigma-Aldrich, Steinheim, Germany) was dissolved in methanol (Sigma-Aldrich, Steinheim, Germany) to prepare a 1,000 ppb Aflatoxin B1 stock solution (stored in freezer). This solution was consecutively diluted with 10 mM PBS (phosphate buffer saline), pH 7.4, generating the appropriate standard solutions (e.g. 10^-5^ – 50 ppb Aflatoxin B1) used in the experiments. The solutions were prepared in the hood and the aflatoxin B1 residues were collected and stocked in special containers since the toxicity of this reagent it is well known. The tracer (Aflatoxin B1 derivative coupled to HRP) was synthesised according to Giersch's protocol [15]. Stock solution of the enzyme tracer was used for daily preparation of the working solution diluting to 1:100 with PBS (10 mM, pH 7.4).

Polyclonal Ig G anti-Aflatoxin B1 antibody (Ab I) was bought from Sigma-Aldrich (Steinheim, Germany). Affinity purified polyclonal Ig G anti-Aflatoxin B1 antibody (Ab II) was kindly provided by Prof. Sergei Eremin (Lomonosov Moscow State University, Russia), while monoclonal Ig G anti-Aflatoxin B1 (Ab III) was developed by Prof. Duck-Hwa Chung's team (Graduate School of Gyeongsang National University, Korea). Protein G and protein A were purchased from Sigma-Aldrich, Steinheim, Germany. Antibodies and protein G/A stock solutions were diluted appropriately with PBS (10 mM, pH 7.4) in order to obtain the proper concentration (200 mg/L) for antibody/protein immobilization on the magnetic beads surface (3 μm diameter, -COOH active groups on the surface, Micromod, Rostock, Germany). The immobilization procedure was performed according with standard protocols mentioned in the previous papers [16, 17]. When the magnetic beads' surface was covered with protein G/A, the antibody was immobilized via affinity interaction. Therefore, the protein G/A-particles were dispersed in the antibody solution. After 30 min (incubation time), the particles were separated from the solution and washed carefully with PBS solution (10 mM, pH 7.4).

Phosphate buffer saline (PBS) was prepared as a stock solution of 100 mM concentration according to the following method: NaCl (80 g), KCl (2 g), Na_2_HPO_4_×2H_2_O (14.3 g) and KH_2_PO_4_ (3.43 g) were dissolved in distilled water (1 l) and the pH was adjusted with either NaOH or HCl. All the reagents were purchased from Merck, Darmstadt, Germany. The stock solution of PBS (100 mM) was diluted with distilled water until reached 10 mM PBS concentration (pH = 7.4). 40 mM citrate buffer solution was prepared by mixing proper volume of 40 mM trisodium citrate solution and 40 mM citric acid solution. The pH value of the buffer was adjusted to 5.5.

The colorimetric substrate solution contained the following reagents: freshly prepared tetramethylbenzidine (TMB, 6 mg/mL in DMSO, Merck, 1.6 ml) and hydrogen peroxide (30 % H_2_O_2_, 14.4 μl) WERE diluted in 40 mM citrate buffer pH 5.5 (100 ml). The substrate reaction was stopped using acidic solution of 5 % H_2_SO_4_. All the reagents were purchased from Sigma-Aldrich, Steinheim, Germany. A classical ELISA reader (Appliskan 2.3, Thermo Fisher Scientific Inc., Waltham, USA) was used for quantifying the analytical signal. Also, the ELISA procedure was performed regarding with the most popular protocol using microtitre plates with 96 wells and 12 channes automatic pipette (Eppendorf).

### 3.2. mp-ELISA Method

The *mp*-ELISA procedure was initiated by dispensing the conjugated-particles (antibody or antibody-protein G/A magnetic beads) suspension (100 μL) in the wells of the microtitre plate (Step 1). The suspension was prepared in 10 mM PBS pH 7.4. A neodymium magnet, placed underneath the ELISA plate provided a magnetic field that fixed the beads at the bottom of the well by its magnetic force. Therefore, the beads were separated from the solution allowing removing the PBS from the well (Step 2). Then, the analyte and/or tracer solution (100 μL) was added to the well (Step 3). The magnet was removed and the plate was gently shaken for 10 min in order to provide a homogeneous content of the reaction well (Step 4). The excess of the analyte and/or tracer were eliminated from the reaction well as indicated based on washing step (Step 5). In this case, the particles were checked with the magnet on the well bottom and the well was washed with PBS solution (10 mM, pH 7.4). Then, the substrate solution (100 μL) was added and incubated for 15 min (Step 6) followed by stopping the coloured reaction with stop solution of 5 % H_2_SO_4_ (50 μL) (Step 7). The analytical signal indicated by ELISA reader was used to quantify the analyte concentration in the sample.

## 4. Conclusions

A new alternative ELISA method (*mp*-ELISA) had been developed for Aflatoxin B1 determination. The new concept uses antibodies immobilized on the surface of magnetic particles as its principle feature. Based on our experiments it was demonstrated that the immobilization procedure apparently “altered” the antibody affinity, even with low amounts of immobilized antibody or improper orientation of the bound antibody on the beads' surface (blocking the antibody active sites). Also, the method performance is closely related with the type of antibody used in the analysis (i.e. any antibody recognized differently the antigen derivative of the tracer structure). As an example, the method sensitivity will be higher if an antibody with higher affinity to the analyte is used. Also, different type of antibodies (e.g. monoclonal, polyclonal, affinity purified polyclonal antibody, *etc.*) will be characterized by different affinity to the antigen and generate different performance of the same method. This was emphasized in our experiments when polyclonal Ig G anti-Aflatoxin B1 antibody (Ab I), affinity purified polyclonal Ig G anti-Aflatoxin B1 antibody (Ab II), and monoclonal Ig G anti-Aflatoxin B1 (Ab III) were separated used for aflatoxin B1 determination based on *mp*-ELISA method. The *mp*-ELISA is one of the most sensitive immuno-methods for Aflatoxin B1 determination (LOD = 1 ppt), to the best of our knowledge. This is a direct consequence of using the magnetic particles as well as the proper anti-Aflatoxin B1 antibody with high affinity for the interested analyte. Immobilization of the antibody on the magnetic particles surface allowed the convenient distribution of the antibody into the volume of the microtiter well improving the efficiency of the immuno-affinity interaction. Therefore, the equilibrium of the immuno-interaction will be achieved faster than in classical ELISA (10 min for *mp*-ELISA), decreasing dramatically the analysis time (maximum 30 min). The *mp*-ELISA involves fewer steps (e.g. the blocking step with BSA was eliminated), larger interface contact liquid-solid, more stable and efficiently bioactive surface (e.g. short incubation time) and lesser immuno-reagents utilised than conventional ELISA, since the antibody is immobilized on the mobile and easy manipulated support (e.g. magnetic particles).

## Figures and Tables

**Table 1.** Influence of antibody immobilization procedure on the *mp*-ELISA characteristics (1:1,000 dilution grade of Ab III-bead suspension and 1:100 dilution grade of tracer).

**Antibody immobilization**		**LOD (ppt)**	**IC50% (ppt)**	**DR (ppt)**
Covalent interaction Ab-bead		1	2	1 - 10
Affinity interaction	Ab-protein G-bead	50	150	80 - 500
	Ab-protein A-bead	100	200	50 - 500

LOD – limit of detection calculated as Aflatoxin B1 concentration, which produced a decrease of the maximum signal (signal for zero dose) of 10 %.

IC50% – inhibition concentration corresponding with Aflatoxin B1 concentration causing 50 % decrease of the maximum signal (signal for zero dose).

DR – dynamic range of analysis calculated as Aflatoxin concentration which provokes a decrease of the zero dose signal in the range 20 – 80 %.
